# Impact of glucokinase activators on the gut microbiota of high-fat diet-induced obese and type 2 diabetic mice

**DOI:** 10.3389/fmicb.2026.1783385

**Published:** 2026-03-19

**Authors:** Lanyu Gao, Qing Shao, Xinyu Zhuo, Yining Xu, Yuwei Zhang

**Affiliations:** 1 Department of Endocrinology and Metabolism, Laboratory of Diabetes and Metabolism Research, West China Hospital, Sichuan University, Chengdu, Sichuan, China; 2 Frontiers Science Center for Disease-Related Molecular Network, West China Hospital, Sichuan University, Chengdu, Sichuan, China; 3 Department of Pharmacy, Institute of Metabolic Diseases and Pharmacotherapy, West China Hospital, Sichuan University, Chengdu, Sichuan, China

**Keywords:** anti-diabetic agents, glucokinase activators, gut microbiota, dorzagliatin, TTP399

## Abstract

**Introduction:**

Glucokinase activators (GKAs) enhance glucose phosphorylation by activating glucokinase (GK) expressed in key metabolic organs (such as the liver, pancreas and intestine), thereby initiating cellular glucose metabolism and contributing to improved glycemic control. Among various GKAs under development, dorzagliatin and cadisegliatin (TTP399) are currently the most promising. The gut microbiota plays a critical role in the pathogenesis and progression of metabolic disorders, including obesity and type 2 diabetes. Substantial evidence indicates that long-term administration of oral glucose-lowering agents, such as metformin, can modulate the composition and function of the gut microbiota. Nevertheless, whether GKAs-as emerging oral hypoglycemic agents-also influence gut microbial homeostasis remains unexplored. This study aims to investigate the effects of oral GKAs on intestinal barrier integrity and gut microbiota composition in high-fat diet (HFD)-induced obese/type 2 diabetic mice. In addition, we compare the differential impacts of distinct GKA agents on glycemic regulation and gut microbial communities.

**Methods:**

Mice were randomly assigned to receive oral gavage of either a vehicle, dorzagliatin, or TTP399 for four consecutive weeks following 5 weeks of HFD feeding. Throughout the study, changes in key metabolic parameters, intestinal barrier integrity, and inflammatory markers were evaluated. Additionally, fecal samples were collected and subjected to 16S-rRNA gene sequencing for analysis of the gut microbiota composition.

**Results:**

Both dorzagliatin and TTP399 exerted beneficial hypoglycemic effects in HFD mice. Furthermore, results also showed that both dorzagliatin and TTP399 regulated the gut microbiota structure in HFD mice, specifically increasing the relative abundance of short-chain fatty acids-producing and anti-inflammatory bacteria. Notably, under the conditions of this study, neither activator exhibited significant effects on intestinal barrier integrity or inflammatory markers.

**Discussion:**

Dorzagliatin and TTP399 are associated with alterations in gut microbiota composition at the genus level in HFD-fed mice, with a concomitant increase in the abundance of beneficial genera, and no significant association with changes in intestinal barrier integrity or inflammation. Further investigation is warranted to elucidate the association between long-term GKAs treatment and microbial communities, as well as the potential relationship between microbial changes and hypoglycemic effects.

## 1 Introduction

Diabetes mellitus is among the most common and serious chronic illnesses, representing a prevalence of 11.1% of the adults, in which type 2 diabetes (T2D) makes up 90% to 95% of all diabetes cases. Without proper management, T2D can cause serious complications such as cardiovascular disease, chronic kidney disease, and retinopathy (Schwarz, 2025). Currently, therapeutic strategies for T2D include lifestyle modifications and medication. While numerous treatments have demonstrated efficacy in glycemic control, some patients still experience challenges such as suboptimal blood glucose regulation or medication-related adverse effects. Consequently, a multitude of novel hypoglycemic agents have been developed and are currently under investigation, including dipeptidyl peptidase-4 inhibitors (DPP-4is), glucagon-like peptide-1 receptor agonists (GLP-1RAs), dual gastric inhibitory polypeptide/glucagon-like peptide-1 receptor agonists (GIP/GLP-1RAs), and G protein-coupled receptor agonists (GPCRs). These agents have demonstrated substantial clinical benefits in improving glycemic control and reducing the risk of complications. Nevertheless, T2D remains a complex metabolic disorder modulated by multiple factors, including genetic susceptibility, environmental factors (e.g., dietary patterns and physical activity levels), and metabolic dysregulation, whose pathophysiological mechanisms have not yet been fully elucidated. Despite the prominent clinical advantages of numerous novel agents, their precise mechanisms of action require further in-depth investigation.

It is well established that the gut microbiota profoundly modulates host metabolic and immune functions. Accumulating evidence indicates that it also contributes to the development and progression of various metabolic disorders, including obesity and T2D (Wu et al., 2021; Fan and Pedersen, 2021). Numerous studies have demonstrated that patients with T2D frequently exhibit gut microbial dysbiosis, and targeted modulation of this microbiota represents a promising strategy for the prevention and treatment of T2D (Gurung et al., 2020; Aw and Fukuda, 2018). Furthermore, gut microbiota dysbiosis and dysfunction can impair intestinal barrier integrity, increasing permeability and promoting the overgrowth of opportunistic pathogens. Disruption of the intestinal barrier further promotes the translocation of gut microbes and harmful metabolic byproducts into the systemic circulation, which in turn impairs glucose metabolism and immune homeostasis, leading to multi-organ damage in the host (Yang et al., 2021; Genser et al., 2018). In a mouse model, it was found that two potentially beneficial species for T2D (*Bacteroides vulgatus* and *B. dorei*) upregulate the expression of tight junction genes in the colon, resulting in reduced intestinal permeability, decreased lipopolysaccharide (LPS) production, and ameliorated endotoxemia (Yoshida et al., 2018). *Akkermansia muciniphila* uses its extracellular vesicles to decrease intestinal permeability; these vesicles enhance tight junctions in the intestine by activating AMP-activated protein kinase (AMPK) in epithelial cells (Chelakkot et al., 2018). Moreover, the outer membrane protein of *A. muciniphila* (Amuc_1100) upregulates the expression of occludin and tight junction protein 1 (Tjp1), thereby improving intestinal barrier integrity (Chelakkot et al., 2018). Additionally, butyrate—a short-chain fatty acid (SCFA) metabolite produced by Faecalibacterium and Roseburia spp.—may further decrease intestinal permeability via the serotonin transporter and peroxisome proliferator-activated receptor-*γ* (PPAR-*γ*) pathways (Kinoshita et al., 2002).

Diabetes mellitus requires long-term pharmacotherapy, with oral administration being the most preferred route due to its superior patient compliance. Several commonly used antidiabetic agents have been shown to improve glycemic control by modulating the gut microbiota composition (Lv et al., 2018), including metformin and DPP-4is. Beyond acting on their respective drug targets to regulate blood glucose levels, these agents—owing to their route of administration—exert direct effects on the gut microbiota, modulate its composition, or alter the intestinal epithelial barrier (Forslund et al., 2015). Fecal samples collected from metformin-treated donors before and 4 months after treatment, when transplanted into germ-free mice, demonstrated improved glucose tolerance in recipients that received microbiota altered by metformin (Wu et al., 2017). Compared to HFD-fed control mice, HFD-fed mice treated with metformin exhibited a higher abundance of the mucin-degrading bacterium Akkermansia spp. Such gut microbial alterations, including an increased population of Akkermansia spp., may contribute to the antidiabetic effects of metformin (Shin et al., 2014). Additionally, DPP-4is have been shown to significantly reverse HFD-induced gut microbial dysbiosis, and these alterations promote glucose homeostasis (Liao et al., 2019). Collectively, these studies indicate that targeted modulation of the gut microbiota composition in patients with T2D may represent an effective therapeutic strategy for disease management. Therefore, investigating the effects of long-term oral hypoglycemic agents on the gut microbiota is of critical clinical and scientific importance.

GKAs represent a class of oral hypoglycemic agents either marketed or under investigation that enhance glucose phosphorylation by activating GK expressed in key metabolic organs (such as the liver, pancreas and intestine). This activation initiates cellular glucose metabolism, thereby contributing to improved glycemic control. GKAs can be classified into dual-action GKAs, which activate both pancreatic and hepatic GK (Grimsby et al., 2003; Eiki et al., 2011; Sarabu et al., 2012), and hepatoselective GKAs (Vella et al., 2019). Among the various GKAs under development, dorzagliatin and TTP399 are currently the most promising. Dorzagliatin was the first GKA approved for clinical use in 2022. It is a dual-acting, orally bioavailable GKA that targets both pancreatic and hepatic GK, enhances GK activity in a glucose-dependent manner, boosts glucose-stimulated insulin secretion, and thus improves glycemic control in T2D patients (Zhu et al., 2022; Yang et al., 2022). TTP399 is a hepatoselective GKA administered orally, which lowers blood glucose by targeting hepatic GK and is currently undergoing Phase III clinical trials (Klein et al., 2021). Nevertheless, whether GKAs-as emerging oral hypoglycemic agents-also influence gut microbial homeostasis remains unexplored. This study aims to investigate the effects of oral GKAs on intestinal barrier integrity and gut microbiota composition in HFD-induced obese and type 2 diabetic mice. In addition, we compare the differential impacts of distinct GKA agents on glycemic regulation and gut microbial communities.

## 2 Materials and methods

### 2.1 Materials and reagents

High-fat diet (HFD) (Research Diets Inc. D12492, 60 kcal% fat) and normal control diet (DIO Series Diets, D12450J) of mice were purchased from CHENGDU DOSSY EXPERIMENTAL ANIMALS CO., LTD. (Chengdu, China). Dorzagliatin (HY-109030) and TTP399 (HY-147254) were purchased from MedChemExpress (MCE, United States). The Mouse Insulin ELISA kit (10–1,247-01) was purchased from Mercodia (Uppsala, Sweden).

### 2.2 Drug dissolution protocol

For dorzagliatin: The vehicle is composed of 10% DMSO +40% PEG300 + 5% Tween-80 + 45% Saline. For TTP399: The vehicle is composed of 15% Cremophor EL + 85% Saline. The dissolution method for dorzagliatin was performed according to the drug solubilization protocol provided by MCE. To maintain a consistent solvent system for both agents, we initially attempted to dissolve TTP399 using the same protocol, however, the solubility was unsatisfactory. Therefore, following the recommended protocol from MCE, TTP399 was finally dissolved in 15% Cremophor EL + 85% saline. Thus, the two drugs used different solvent systems, which was the primary reason for establishing two independent control groups in the study.

### 2.3 Animal study

**Figure 1:**
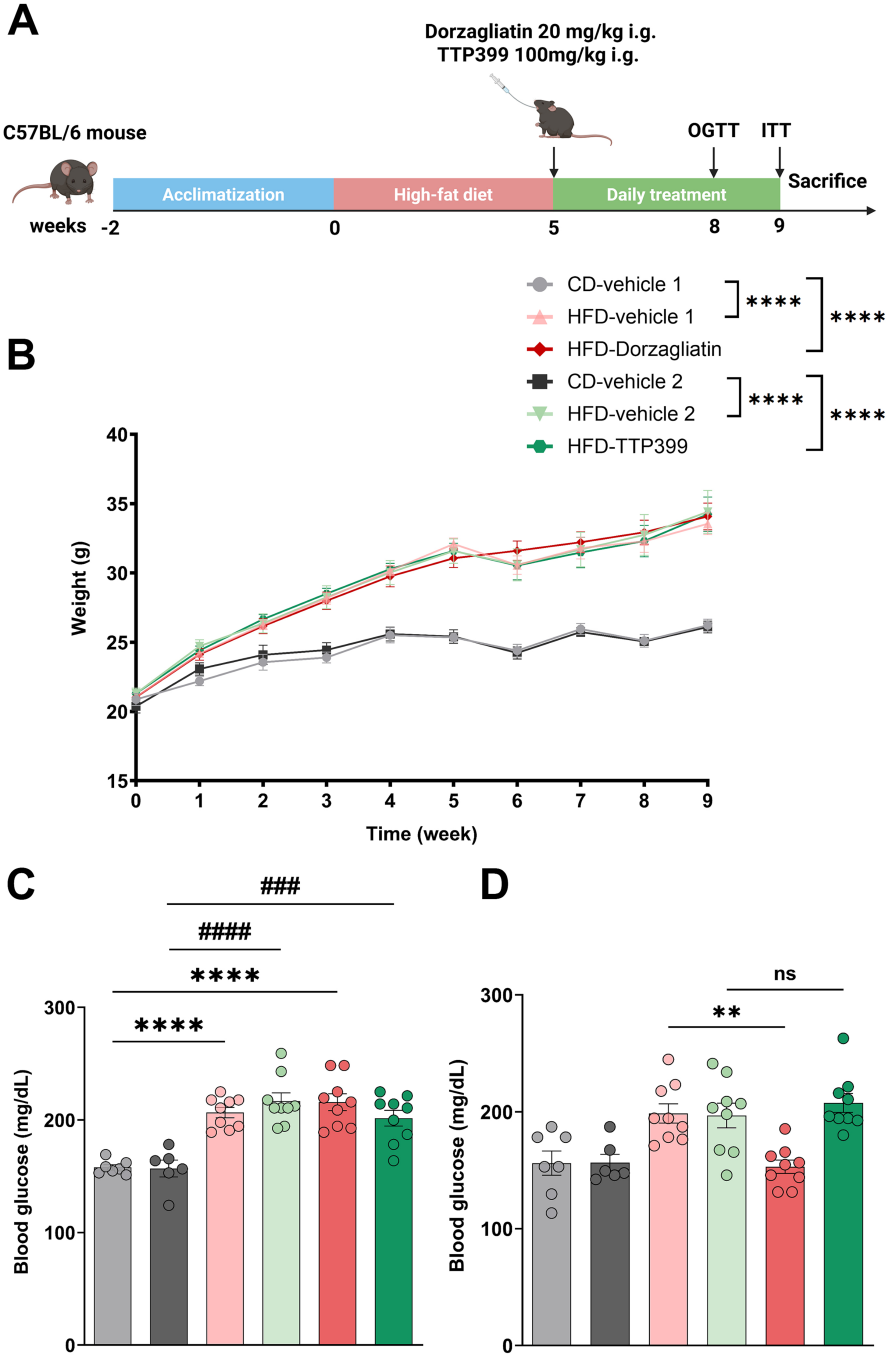
**(A)** Experimental design; **(B)** body weight; **(C)** FBG levels before treatment started (at 5 weeks); **(D)** FBG levels after treatment (at 9 weeks). All data are presented as mean ± SE (CD_Vehicle1 group: *n* = 7, CD_Vehicle2 group: *n* = 6, HFD_Vehicle1 group: *n* = 9, HFD_Vehicle2 group: *n* = 9, HFD_Dorzagliatin group: *n* = 9, HFD_TTP399 group: *n* = 9). One-way or two-way ANOVA followed by Tukey’s multiple comparison test was used to determine significant differences. ^****^ *p* < 0.0001 (CD_Vehicle1 vs. HFD_Vehicle1, CD_Vehicle1 *vs.* HFD_Dorzagliatin), ^**^p < 0.01 (HFD_Vehicle1 vs. HFD_Dorzagliatin), ^####^*p* < 0.0001 (CD_Vehicle2 *vs.* HFD_Vehicle2), ^###^*p* < 0.001 (CD_Vehicle2 *vs.* HFD_TTP399).

A total of 54 male C57BL/6 J mice (8 weeks old) were purchased from GemPharmatech Co., Ltd. (Chengdu, China), weighing 18–22 g. All mice were housed in a temperature-controlled room (22–25 °C) under specific pathogen-free (SPF) conditions with a standard 12 h light/dark cycle. After a 2-week acclimatization period, mice were fed a HFD (HFD groups, 60% fat, 20% protein, 20% carbohydrate (kcal/100 g)) or a normal control diet (CD groups) for 5 weeks (*n* = 9 per group). Then these mice were administered 20 mg/kg of dorzagliatin (HFD_Dorzagliatin group), 100 mg/kg of TTP399 (HFD_TTP399 group), or vehicle for dorzagliatin dissolution (CD_Vehicle1 group and HFD_Vehicle1 group), or vehicle for TTP399 dissolution (CD_Vehicle2 group and HFD_Vehicle2 group) in the volume equivalent to dorzagliatin or TTP399 once daily by gavage for 4 weeks. All mice had free access to water and food throughout the experiment, and their body weight was monitored weekly during the experiment, and the fasting blood glucose (FBG) levels were also measured every week during the treatment period (Figure 1A). Due to accidental death caused by gavage injury and hypoglycemia during the ITT, some mice were excluded from the final analysis. The final valid sample sizes were as follows: CD_Vehicle1 group: *n* = 7, CD_Vehicle2 group: *n* = 6, HFD_Vehicle1 group: *n* = 9, HFD_Vehicle2 group: *n* = 9, HFD_Dorzagliatin group: *n* = 9, HFD_TTP399 group: *n* = 9.

All experimental procedures were approved by the Animal Ethics Committee of West China Hospital, Sichuan University (Approval No. 20250528004).

### 2.4 Oral glucose tolerance test (OGTT) and insulin tolerance test (ITT)

All mice were fasted overnight for OGTT after 3 weeks of treatment, and then administered glucose at 2 g/kg of body weight by oral gavage. Tail blood glucose concentrations were measured at 0 (fasting glucose, taken before glucose gavage), 15, 30, 60, 90 and 120 min after glucose gavage using the Bayer glucometer. The ITT was performed 2–3 days before sacrifice (after 4 weeks of treatment). After 6 h of fasting, and then administered insulin at 0.75 IU /kg of body weight via intraperitoneal (i.p.) injection. Tail blood glucose concentrations were measured at 0 (fasting glucose, taken before insulin injection), 15, 30, 60, 90 and 120 min after insulin injection using the Bayer glucometer. The glucose level was then plotted against time, and then calculated the area under the glucose curve (AUC) of the glucose concentration by the trapezoidal rule. Insulin secretion levels were measured at 0 min during the ITT using a Mouse Insulin ELISA kit. The homeostasis model assessment of insulin resistance (HOMA-IR) was calculated as previously reported: HOMA-IR = [fasting glucose (mmol/l) × fasting insulin (mU/l)]/22.5 (Matthews et al., 1985).

### 2.5 Intestinal barrier integrity and inflammation

The duodenum, jejunum, ileum, and colon were harvested after sacrifice and frozen at −80 °C. Total RNA was isolated from the mouse intestinal tissues with RNAiso Plus (TAKARA, Code No. 9108/9109) and reverse-transcribed using PrimeScript™ FAST RT reagent Kit with gDNA Eraser (TAKARA, Code No. RR092A), and subjected to quantitative polymerase chain reaction (qPCR) using TB Green® Premix Ex Taq™ II (TAKARA, Code No. RR820A). The thermal cycling conditions were as follows: 95 °C for 30 s, 40 cycles at 95 °C for 5 s and 60 °C for 30 s, followed by 95 °C for 15 s, 60 °C for 1 min, and 95 °C for 15 s. The mRNA expression levels of Tjp1, occludin, claudin-1, TNF-*α*, IL-1*β*, and IL-6 were quantitatively analyzed and normalized to 18S levels. The forward and reverse primer sequences are provided in Supplementary Table S1.

### 2.6 Histological staining

Appropriate lengths of the ileum and colon tissues were fixed with 4% paraformaldehyde (PFA). The samples were embedded in paraffin and sliced. The slices were placed on a glass slide (polylysine coated) followed by dewaxing and rehydration. Haematoxylin and eosin (H&E) staining was carried out according to the manufacturers’ instructions followed by microscopic examination for pathological analysis (Nikon, Tokyo, Japan). The histological scores, including crypt structure, inflammation, mucosal thickening, crypt abscesses, and goblet cell exhaustion, were assessed as shown in Table 1 (Zhao et al., 2024).

**Table 1:** Scoring criteria for histopathology.

Score	Crypt architecture	Degree of inflammatory cell infiltration	Mucosal thickening	Crypt abscess	Goblet cell depletion
0	Normal	Normal	Base of crypt sits on the muscularis mucosae	Absent	Absent
1	Mild crypt distortion	Mild	Mild muscle thickening	Present	Present
2	Moderate crypt distortion	Moderate	Moderate muscle thickening	—	—
3	Severe crypt distortion with loss of entire crypts	Dense inflammatory infiltrate	Marked muscle thickening	—	—

### 2.7 Analysis of gut microbiome by 16S-rRNA gene sequencing

After the treatment, fecal samples from all mice were collected in 1.5 mL Eppendorf tubes. During the collection of feces, all the animals were kept in a single cage to avoid intragroup cross-contamination. Samples were kept on ice and then stored at −80 °C until further analysis. Fecal genomic DNA was extracted from 0.1 g frozen fecal samples using the TIANamp Stool DNA Kit (TIANGEN BIOTECH (BEIJING) CO., LTD) according to the manufacturer’s protocol. DNA quality was detected by 1% agarose gel electrophoresis. Qualified DNA samples were diluted with sterile nuclease-free water, to a final concentration of 1 ng/μL for downstream experiments. The diluted genomic DNA was used as a template for PCR amplification of the target 16S rRNA gene region using barcode-tagged primers. PCR products were verified by 2% agarose gel electrophoresis and subsequently purified using magnetic bead-based purification kits to remove primer dimers and non-specific amplification products. A small-fragment library was constructed based on the characteristics of the amplified region, and sequencing was performed using the Illumina NovaSeq platform. Following read assembly and quality filtering, amplicon sequence variants (ASVs) were denoised, and the resulting valid data were subjected to taxonomic annotation and abundance analysis (Detailed information on quality control parameters for each sample can be found in the Supplementary materials, with the corresponding file name “RawData/CleanData”). The construction of the high-throughput sequencing library and Illumina NovaSeq sequencing were conducted by Novogene (Chengdu, China). All data analyses were performed using NovoMagic (https://magic-plus.novogene.com/). All fecal samples were processed and sequenced in a single batch to minimize batch effects. In addition, mice from different treatment groups were housed in a mixed-cage manner to reduce potential cage effects.

### 2.8 Statistical analysis

Statistical analysis was performed using GraphPad Prism software (version 10.4.0). All data are presented as mean ± standard error (SE). One-way or two-way analysis of variance (ANOVA) followed by Tukey’s multiple comparison test was used to determine significant differences. Grubbs’ test, also called the extreme studentized deviate (ESD) method, was used to exclude significant outliers. The alpha-diversity indices of gut microbiota (including Observed-features and Chao1) were analyzed using the Kruskal-Wallis test. Spearman’s correlation analyses were performed using SPSS software (version 29.0.1.0). A *p* value < 0.05 was considered statistically significant. The error discovery rate (FDR) was managed via the Benjamin-Hochberg (BH) method, where an FDR value below 0.25 served as the threshold for determining significant differences.

## 3 Results

### 3.1 Effects of GKAs on body weight and glucose metabolism in HFD-fed mice

Mice in the CD groups maintained normal body weight, while those in the HFD groups exhibited a significant weight gain. Neither dorzagliatin nor TTP399 treatments affected body weight (Figure 1B). At 5 weeks, before treatment started, FBG levels were higher in the HFD groups compared to the CD groups (Figure 1C). By 9 weeks, after treatment, mice treated with dorzagliatin had lower FBG levels than the HFD_Vehicle1 group. Dorzagliatin treatment reduced FBG levels in HFD-mice, whereas TTP399 did not (Figure 1D).

**Figure 2:**
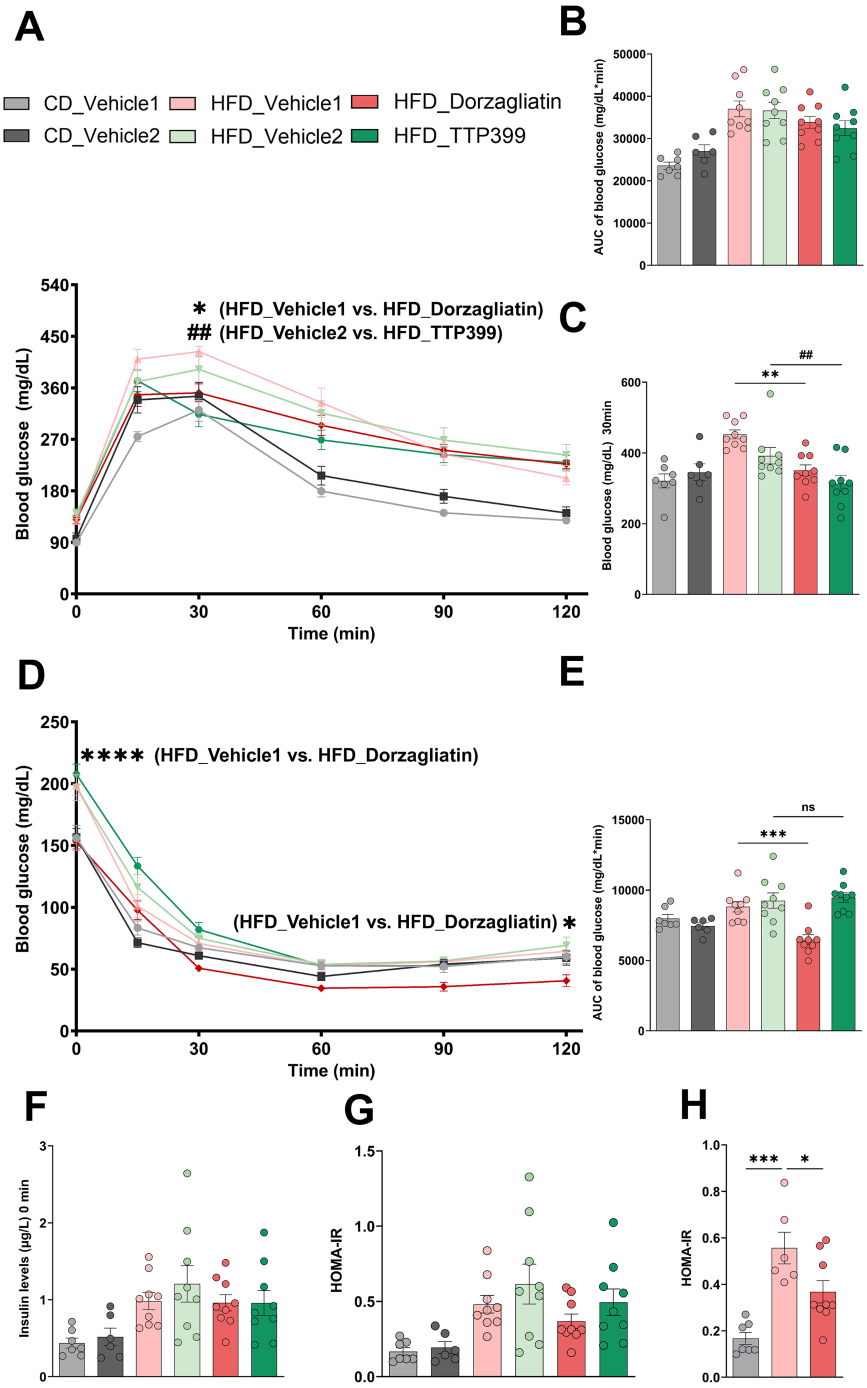
Effects of dorzagliatin and TTP399 on glucose tolerance and insulin tolerance in HFD-fed mice. **(A)** OGTT after 3 weeks of treatment; **(B)** AUC calculated from the glucose concentrations versus time for the period of 0–120 min after glucose load by the trapezoidal rule; **(C)** Blood glucose levels at 30 min post-administration during the OGTT; **(D)** ITT after 4 weeks of treatment; **(E)** AUC of the ITT; **(F)** Fasting insulin (FINS) levels and HOMA-IR value **(G,H)** during the ITT. Values are expressed as mean ± SE (CD_Vehicle1 group: *n* = 7, CD_Vehicle2 group: *n* = 6, HFD_Vehicle1 group: *n* = 9, HFD_Vehicle2 group: *n* = 9, HFD_Dorzagliatin group: *n* = 9, HFD_TTP399 group: *n* = 9). Differences were assessed by one-way ANOVA or two-way ANOVA. ^****^p < 0.0001 (HFD_Vehicle1 vs. HFD_Dorzagliatin), ^***^p < 0.001 (E: HFD_Vehicle1 vs. HFD_Dorzagliatin, H: CD_Vehicle1 *vs.* HFD_Vehicle1), ^**^p < 0.01 (HFD_Vehicle1 vs. HFD_Dorzagliatin), ^*^p < 0.05 (HFD_Vehicle1 *vs.* HFD_Dorzagliatin), ^##^ *p* < 0.01(HFD_Vehicle2 *vs.* HFD_TTP399).

In the OGTT after 3 weeks of treatment, before glucose administration, blood glucose levels were higher in the HFD groups than in the CD groups. 15 minutes after glucose was given, blood glucose levels spiked across all six groups (Figures 2A,B). At 30 min post-administration, blood glucose levels in the dorzagliatin and TTP399 groups were significantly lower than in the Vehicle groups, indicating these agents partially restored glucose regulation in the mice (Figure 2C). In the ITT after 4 weeks of treatment, the HFD_Dorzagliatin group showed lower blood glucose levels compared to the HFD_Vehicle1 group (Figures 2D,E). We measured fasting insulin levels of mice in the ITT (Figure 2F), and calculated the HOMA-IR value (Figures 2G,H). The results showed that compared to the HFD_Vehicle1 group, the HOMA-IR value of the HFD_Dorzagliatin group was significantly decreased, suggesting that dorzagliatin partially improved insulin resistance in HFD-fed mice. In contrast, TTP399 had no notable effect on HFD-induced insulin resistance (Figure 2G).

### 3.2 Effects of GKAs on the composition of the gut microbiota in HFD-fed mice

**Figure 3:**
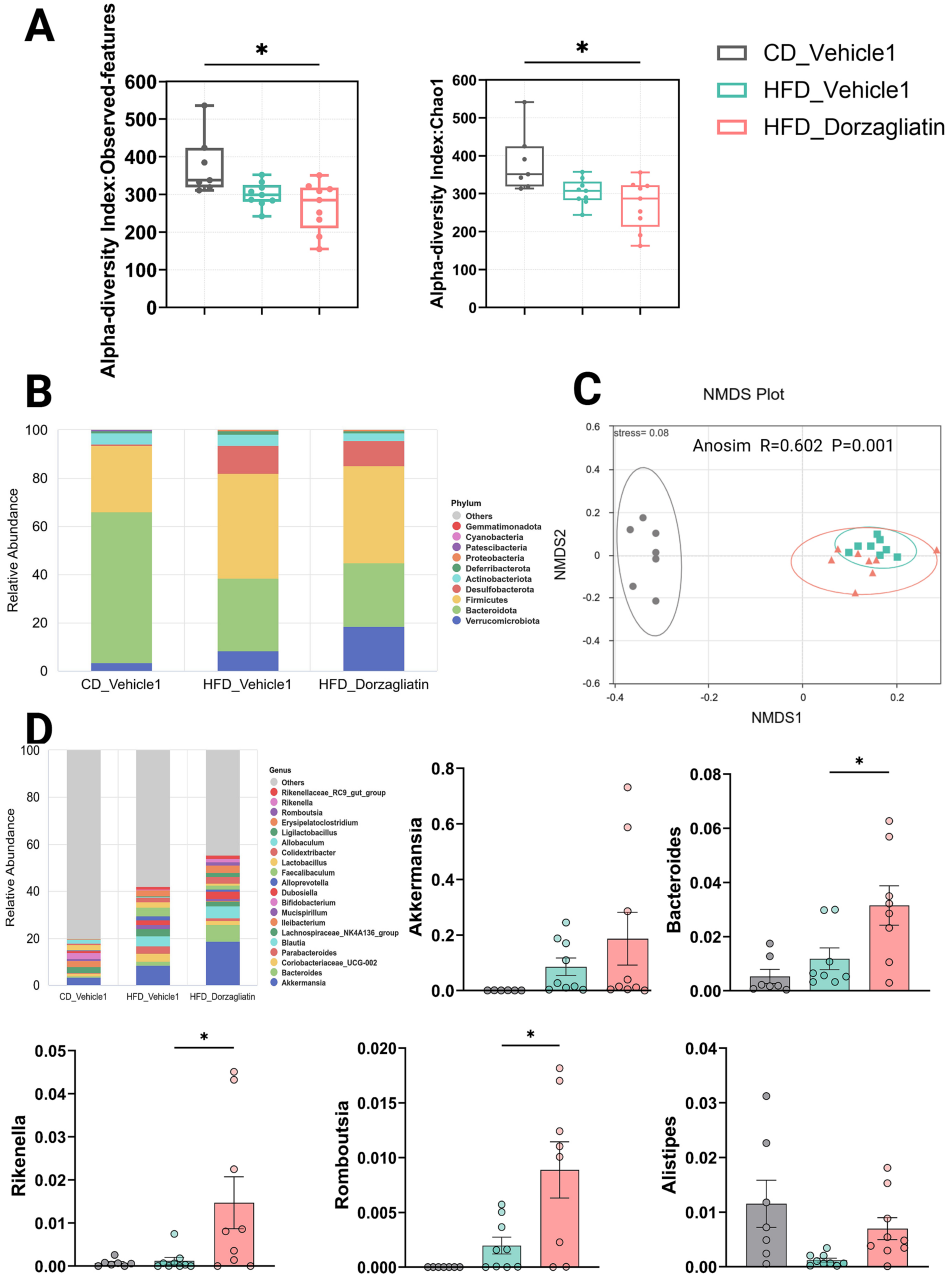
Effects of dorzagliatin on the composition of the gut microbiota in HFD-fed mice. **(A)** Alpha diversity analysis of observed-features index and Chao1 index were analyzed using the Kruskal-Wallis test; **(B)** Relative abundance of the gut microbiota at the phylum levels; **(C)** NMDS analysis plot of the gut microbiota based on unweighted-Unifrac distance; **(D)** Relative abundance of the gut microbiota at the genus levels. Values are expressed as mean ± SE (CD_Vehicle1 group: *n* = 7, CD_Vehicle2 group: *n* = 6, HFD_Vehicle1 group: *n* = 9, HFD_Vehicle2 group: *n* = 9, HFD_Dorzagliatin group: *n* = 9, HFD_TTP399 group: *n* = 9). Differences were assessed by one-way ANOVA.

As the number of observed species increased, the rarefaction curves, species abundance curves, and box plots gradually reached a plateau, indicating sufficient and consistent sampling, appropriate sequencing depth, and the suitability of the data for further analysis (Supplementary Figures S1, S2). To assess the gut microbiota diversity, two alpha-diversity indices were calculated, including observed-features index and Chao1 index. The analysis showed that HFD feeding decreases gut microbiota diversity (Figure 3A), consistent with a previous study (Mamun et al., 2025), but there is no significant function regulating the alpha diversity with the addition of dorzagliatin. In the Beta diversity analysis, the non-metric multidimensional scaling (NMDS) plot based on unweighted UniFrac distances showed significant differences in gut bacterial composition, with the gut microbiota of CD group distinctly separated from that of HFD group (Figure 3C). To further investigate the impact of dorzagliatin on the gut microbiota in HFD-fed mice, we analyzed the relative abundance of gut microbiota at the phylum and genus levels, at the phylum level, Bacteroidetes was the predominant phylum in the CD_Vehicle1 group. In contrast, both the HFD_Vehicle1 and HFD_Dorzagliatin groups showed increased levels of Firmicutes and decreased levels of Bacteroidetes (Figure 3B; Supplementary Figure S3A), which is consistent with the gut microbiota dysbiosis caused by HFD (Turnbaugh et al., 2006). At the genus level, the results showed that compared to the HFD_Vehicle1 group, the HFD_Dorzagliatin group increased relative abundance of several bacterial genera, including Akkermansia, Bacteroides, Rikenella, Romboutsia, and Alistipes (Figure 3D; Supplementary Figure S3B). To explore specific differences in the microbiota among groups, we used Linear discriminant analysis effect size (LEfSe) to identify the altered bacterial taxa. Results are shown as a cladogram and LDA histograms (LDA score > 4), which illustrate the taxonomic classification and hierarchical distribution of gut microbial communities in each group, along with the significantly enriched species and their relative importance (Supplementary Figure S3C).

**Figure 4:**
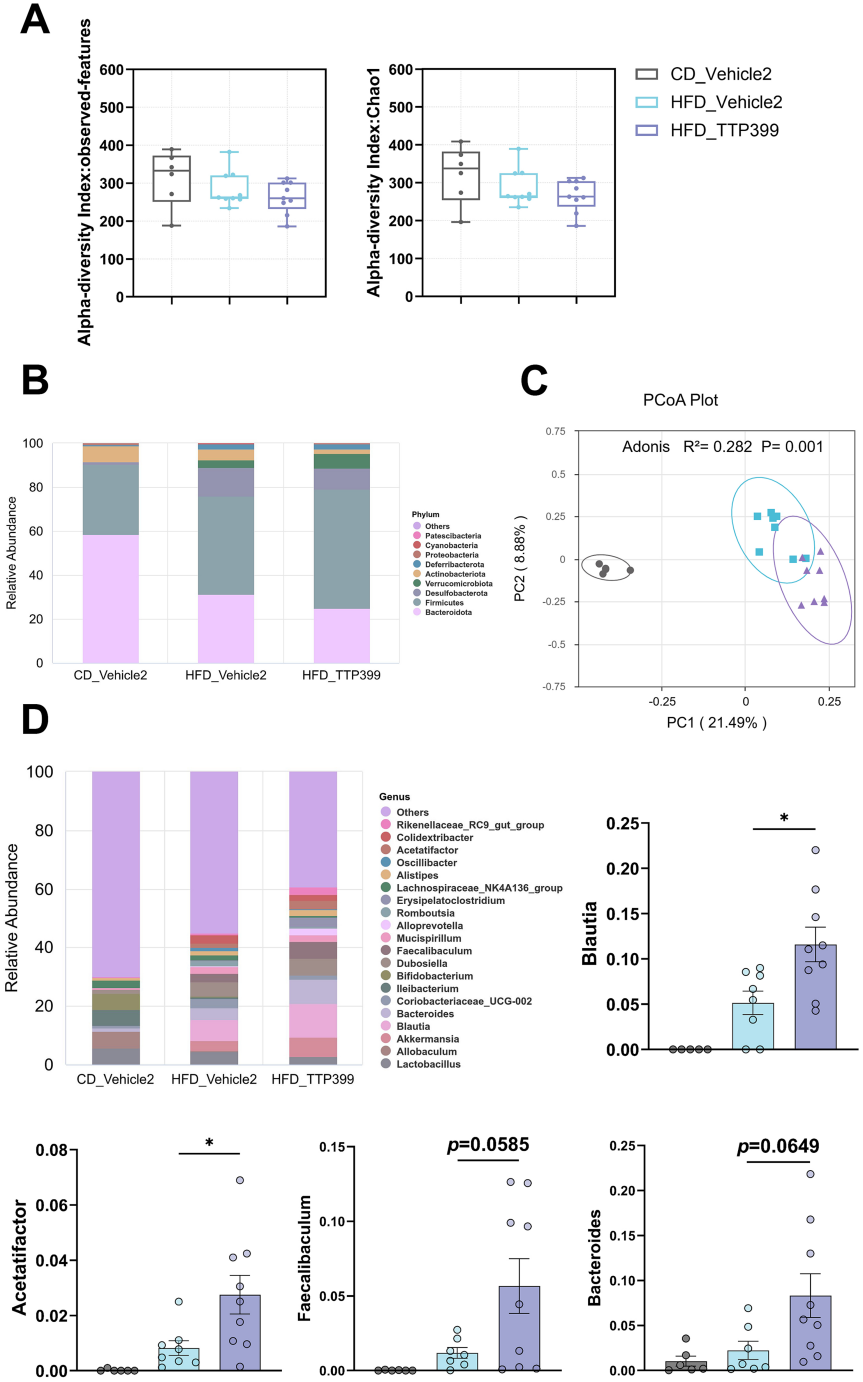
Effects of TTP399 on the composition of the gut microbiota in HFD-fed mice. **(A)** Alpha diversity analysis of observed-features index and Chao1 index were analyzed using the Kruskal-Wallis test; **(B)** Relative abundance of the gut microbiota at the phylum levels; **(C)** PCoA analysis plot of the gut microbiota based on Jaccard distances; **(D)** Relative abundance of the gut microbiota at the genus levels. Values are expressed as mean ± SE (CD_Vehicle1 group: *n* = 7, CD_Vehicle2 group: *n* = 6, HFD_Vehicle1 group: *n* = 9, HFD_Vehicle2 group: *n* = 9, HFD_Dorzagliatin group: *n* = 9, HFD_TTP399 group: *n* = 9). Differences were assessed by one-way ANOVA. ^*^p < 0.05.

In the Alpha diversity analysis, using the observed-features index and Chao1 index, it was found that gut microbiota diversity was lower in both the HFD_Vehicle2 and HFD_TTP399 groups compared to the CD_Vehicle2 group (Figure 4A), TTP399 treatment had no significant effect on Alpha diversity, and for Beta-diversity analysis, principal coordinates analysis (PCoA) plots based on Jaccard distances also demonstrated a significant separation in gut microbiota composition between CD group and HFD group(Figure 4C). Moreover, TTP399 treatment also caused shifts in the gut microbiota at both the phylum and genus levels. At the phylum level, both the HFD_Vehicle2 and HFD_TTP399 groups also showed increased levels of Firmicutes and decreased levels of Bacteroidetes, which is consistent with the gut microbiota dysbiosis caused by HFD (Figure 4B; Supplementary Figure S4A). At the genus level, the analysis showed that TTP399 treatment increased the levels of bacteria genera Blautia, Acetatifactor, Faecalibaculum, and Bacteroides. (Figure 4D; Supplementary Figure S4B). To further explore differences between groups, we also conducted LEfSe analysis. The findings are displayed as a cladogram and LDA histograms (LDA score > 4), highlighting the taxonomic hierarchy of gut microbiota in each group, along with the notably enriched species and their relative significance (Supplementary Figure S4C).

### 3.3 GKAs exert no significant effects on intestinal barrier integrity or inflammation in HFD-fed mice

**Figure 5:**
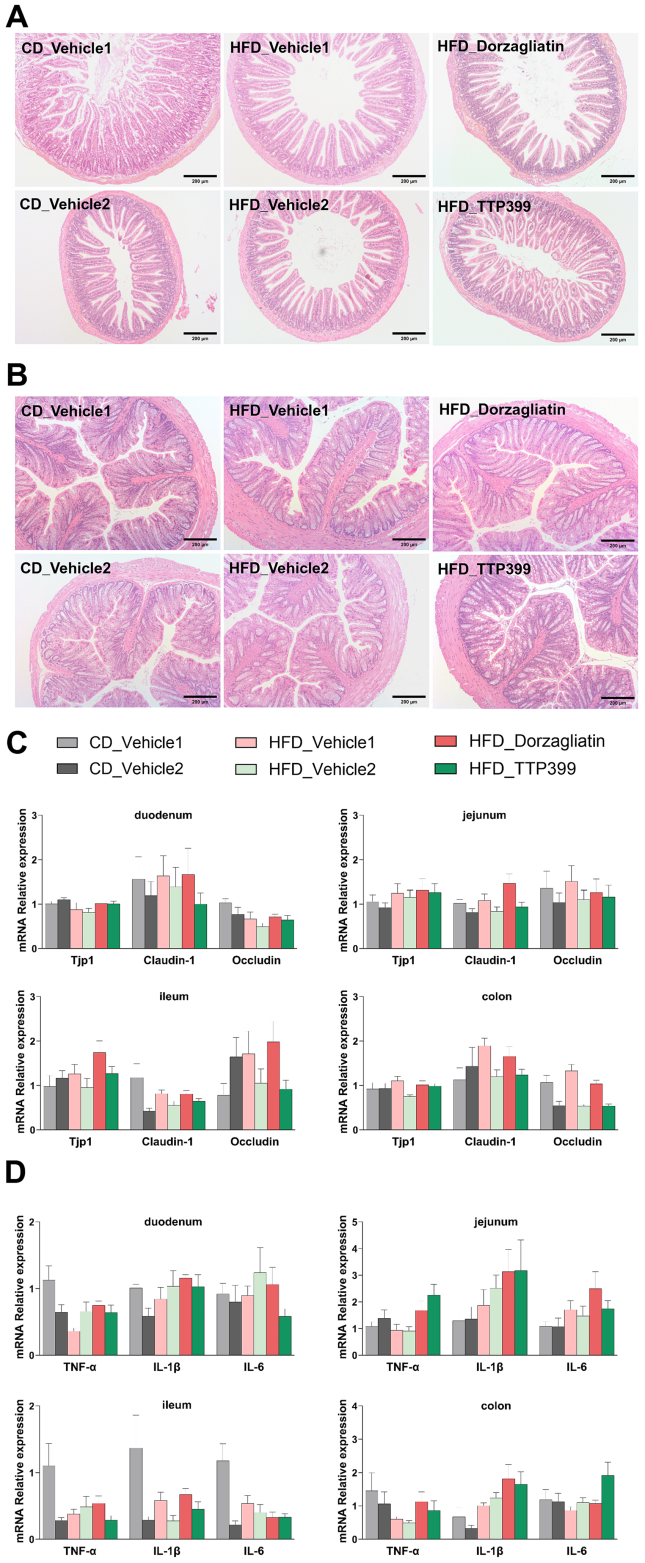
Dorzagliatin and TTP399 have no significant effect on intestinal barrier integrity or inflammation in HFD-fed mice. **(A)** HE staining results of ileum; **(B)** HE staining results of colon; **(C)** Expression levels of Tjp1, claudin-1, and occludin in the duodenum, jejunum, ileum, and colon after 4 weeks of treatment; **(D)** Expression levels of TNF-*α*, IL-1β, and IL-6 in the duodenum, jejunum, ileum, and colon after 4 weeks of treatment. Values are expressed as mean ± SE (CD_Vehicle1 group: *n* = 7, CD_Vehicle2 group: *n* = 6, HFD_Vehicle1 group: *n* = 9, HFD_Vehicle2 group: *n* = 9, HFD_Dorzagliatin group: *n* = 9, HFD_TTP399 group: *n* = 9). Differences were assessed by one-way ANOVA.

The intestinal histopathology was evaluated by H&E staining, the tissue structures of the ileum and colon of six groups of mice were intact. The crypt structures of ileum and colon remained relatively intact, with preserved intestinal mucosa and glands, and no significant inflammatory cell infiltration, crypt abscesses, or goblet cell loss was observed (Figures 5A,B). Histological scores showed no significant differences among groups (Supplementary Figures S5A,B). This result demonstrated that oral administration of dorzagliatin and TTP399 does not induce intestinal damage in HFD mice.

Disruption of the gut microbiota can weaken the intestinal barrier, leading to the breakdown of tight junctions, increased intestinal permeability, and the passage of microbial metabolites (e.g., LPS) into the bloodstream, which triggers metabolic endotoxemia. Intestinal permeability is regulated by tight junctions, with Tjp1, Occludin, and Claudin-1 being key proteins of intestinal epithelial tight junctions. These proteins are crucial for maintaining the integrity of the intestinal barrier, controlling permeability, and communicating with the gut microbiota (Genser et al., 2018; Cani et al., 2009; Cani et al., 2008). We assessed the expression levels of Tjp1, Occludin, and Claudin-1 in the duodenum, jejunum, ileum, and colon of mice using qRT-PCR. The results showed that neither dorzagliatin nor TTP399 significantly affected the expression of these genes (Figure 5C). To further investigate the effects on intestinal inflammation in HFD-fed mice, we measured TNF-*α*, IL-1*β*, and IL-6 levels in the same intestinal segments. (Figure 5D). Notably, in this study, neither activator exhibited significant effects on intestinal barrier integrity or inflammatory markers.

### 3.4 Correlation analysis of metabolic parameters, intestinal barrier integrity, and gut microbiota

**Figure 6:**
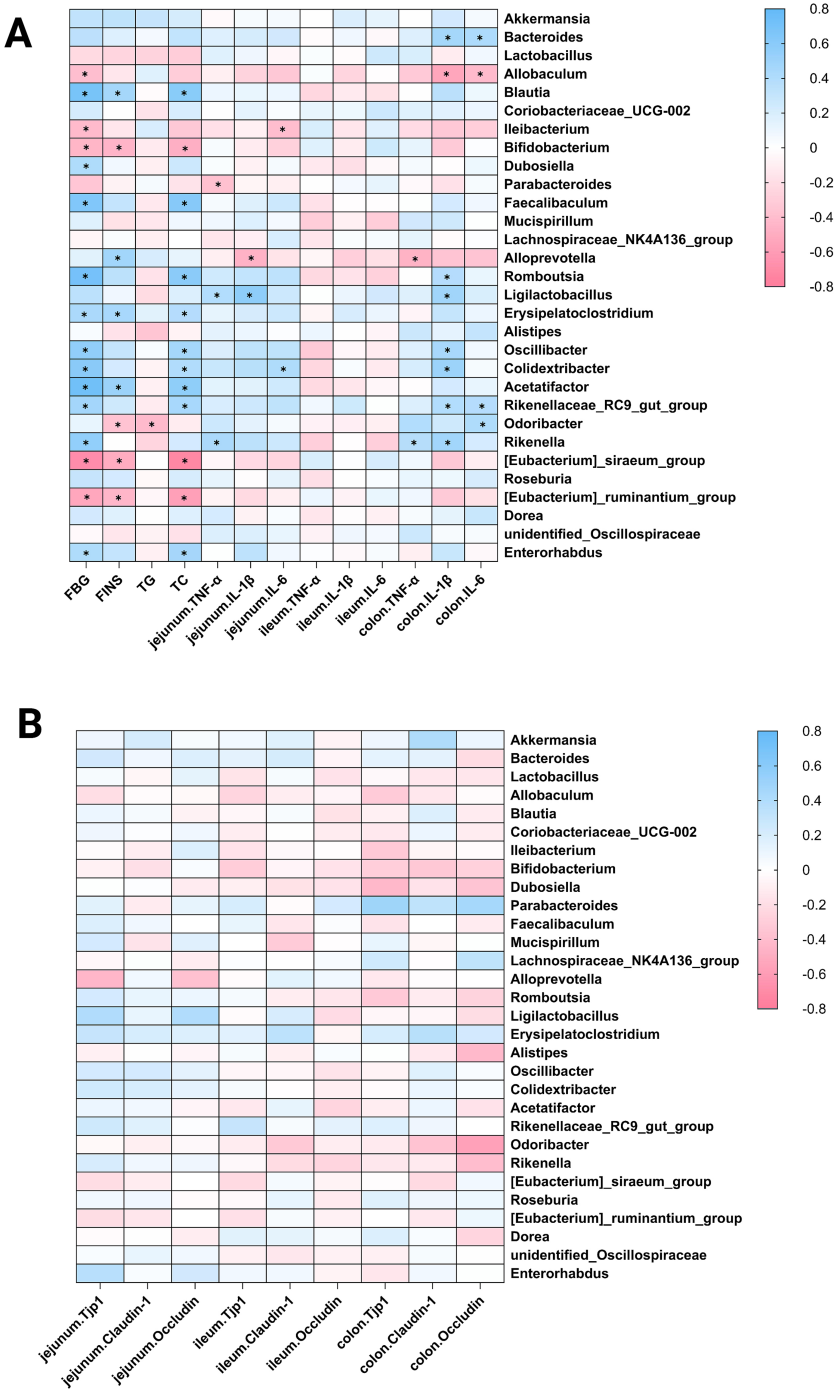
Correlation analysis. **(A)** Correlation analysis between biochemical indices, inflammatory markers, and gut microbiota; **(B)** Correlation analysis between intestinal barrier integrity and gut microbiota. (CD_Vehicle1 group: *n* = 7, CD_Vehicle2 group: *n* = 6, HFD_Vehicle1 group: *n* = 9, HFD_Vehicle2 group: *n* = 9, HFD_Dorzagliatin group: *n* = 9, HFD_TTP399 group: *n* = 9).

To explore the relationships among metabolic parameters, tight junction proteins, intestinal inflammation factors, and gut microbiota, we conducted a Spearman correlation analysis. The results showed that FBG and FINS were significantly positively correlated with the relative abundance of Blautia, Erysipelatoclostridium, and Acetatifactor. Conversely, they were significantly negatively correlated with Bifidobacterium, [Eubacterium]_siraeum_group, and [Eubacterium]_ruminantium_group. Additionally, FBG was significantly positively associated with Dubosiella, Faecalibaculum, Romboutsia, Oscillibacter, Colidextribacter, Rikenellaceae_RC9_gut_group, and Enterorhabdus, and negatively associated with Allobaculum and Ileibacterium. Moreover, FINS was significantly positively correlated with Alloprevotella, and negatively associated with Odoribacter. Triglyceride (TG) levels showed a negative correlation with Odoribacter. Total cholesterol (TC) was significantly positively correlated with Blautia, Faecalibaculum, Romboutsia, Erysipelatoclostridium, Oscillibacter, Colidextribacter, Acetatifactor, Rikenellaceae_RC9_gut_group, and Enterorhabdus, and negatively correlated with Bifidobacterium, [Eubacterium]_siraeum_group, and [Eubacterium]_ruminantium_group. Indices of intestinal inflammation were strongly positively linked to the relative abundance of Bacteroides, Romboutsia, Ligilactobacillus, Oscillibacter, Colidextribacter, Rikenellaceae_RC9_gut_group, Odoribacter, and Rikenella. Conversely, they were significantly negatively correlated with Allobaculum, Ileibacterium, and Alloprevotella (Figure 6A). Furthermore, there was no significant correlation between intestinal tight junction protein markers and these bacterial genera (Figure 6B).

## 4 Discussion

GKAs exert hypoglycemic effects by enhancing GK sensitivity to glucose. To date, no studies have investigated how this novel class of antidiabetic agents modulates the gut microbiota. This study is the first to explore the effects of two emerging antidiabetic drugs—dorzagliatin and TTP399—on intestinal barrier integrity, intestinal inflammation, and gut microbial composition, and we compare the differential impacts of distinct GKA agents on glycemic regulation and gut microbial communities.

It is noteworthy that while both dorzagliatin and TTP399 improved glucose tolerance in HFD mice, only dorzagliatin reduced FBG and improved insulin resistance. Consistent with previous reports, dorzagliatin improves pancreatic *β*-cell function and insulin resistance (Yang et al., 2022). In contrast, studies on TTP399 have primarily highlighted its hypoglycemic effects, lack of hypoglycemia induction, and absence of weight gain, without confirming its ability to improve peripheral tissue insulin sensitivity (Vella et al., 2019). Our findings also indicate that TTP399 does not alleviate insulin resistance. Dorzagliatin is a dual-acting GK activator targeting both pancreatic and hepatic GK. In the pancreas, it enhances glucose-stimulated insulin secretion (GSIS) and improves *β*-cell function. In the liver, it promotes dissociation of the GK-GKRP complex, facilitating GK translocation to the cytoplasm, thereby increasing hepatic glucose uptake and glycogen synthesis (Haddad et al., 2024). Dorzagliatin also stimulates GLP-1 secretion (Chen et al., 2023). In contrast, TTP399 is a liver-selective GK activator specifically targeting hepatic GK without affecting pancreatic GK. TTP399 directly activates GK without disrupting the GK-GKRP complex, enhancing the enzyme’s catalytic activity, but does not enhance GSIS or directly improve pancreatic β-cell function. The lack of pancreatic target effects for TTP399 may explain its absence of significant impact on fasting glucose and insulin resistance, as these parameters largely depend on basal insulin secretion and peripheral insulin sensitivity. Therefore, we speculate that this discrepancy may be attributed to their distinct tissue-specific mechanisms of action.

The HFD can reduce gut microbial diversity. Our analyses of alpha-diversity and beta -diversity suggest that variations in microbial diversity within and between samples are mainly influenced by HFD, rather than by dorzagliatin or TTP399. The gut microbiota’s two predominant bacterial phyla are Firmicutes and Bacteroidetes. Research indicates that a HFD tends to decrease Bacteroidetes and increase Firmicutes abundance (Murphy et al., 2015). An obesity study found higher levels of Firmicutes and lower Bacteroidetes, which aligns with our findings in HFD-fed mice, showing increased Firmicutes and reduced Bacteroidetes (Ley et al., 2006). Our results support the idea that dietary differences primarily drive the observed variations in microbial diversity within and between samples.

In some studies, individuals on HFD show reduced gut bacterial diversity, with simultaneous shifts at the family, genus, or species level—changes that may be more biologically significant than changes in the Firmicutes/Bacteroidetes ratio (Aguirre and Venema, 2015). Therefore, to further investigate the effects of dorzagliatin and TTP399 on the gut microbiota of HFD-fed mice, we analyzed the gut microbiota composition at the genus level. The results revealed that dorzagliatin substantially increased the relative abundance of Akkermansia, Bacteroides, Rikenella, Romboutsia, and Alistipes. TTP399 significantly increased the relative abundance of Blautia, Acetatifactor, Faecalibaculum, and Bacteroides. Akkermansia has been reported to be associated with improvements in obesity, type 1 diabetes (T1D), and T2D in mice, and is recognized as a next-generation probiotic with anti-inflammatory properties and the capacity to modulate metabolic process disorders (Everard et al., 2013; Plovier et al., 2017; Cani et al., 2022). Akkermansia also has been reported to be associated with enhanced intestinal barrier function, decreases LPS translocation, and provides metabolic protection, and is correlated with improved insulin sensitivity and glucose tolerance through its anti-inflammatory mechanisms. Akkermansia is also associated with alleviated glucose metabolic disorders by reducing the endoplasmic reticulum (ER) stress, while reducing lipid accumulation in the liver and skeletal muscle, which are correlated with improved insulin sensitivity (Ke et al., 2021; Sanjiwani et al., 2022). Bacteroides species can digest complex carbohydrates and produce SCFAs like acetate and butyrate, which serve as energy sources for the host and help maintain intestinal health under certain conditions. *Bacteroides vulgatus* has been shown to be associated with reduced HFD-induced obesity in mice by regulating intestinal serotonin production and lipid absorption (Wen et al., 2024). Rikenella, a potential probiotic, plays a crucial role in maintaining metabolic balance, supporting immune function, and promoting gut health. It ferments propionate to generate energy in normal cells, stimulates gluconeogenesis, and ferments carbohydrates (e.g., glucose) to support its growth. Romboutsia shows a strong positive correlation with linoleic acid. Romboutsia lituseburensis has been demonstrated to regulate lipid metabolism, including triglyceride and cholesterol processing. Additionally, Romboutsia can influence lipid metabolism, resulting in the production of unsaturated fatty acids and antimicrobial peptides, thereby boosting the host’s ability to resist pathogen invasion (Zhang et al., 2024). Blautia, a common intestinal acetate-producing bacterium, has been associated with the regulation of insulin signaling and fat accumulation in adipocytes, potentially via activation of G protein-coupled receptors GPR41 and GPR43. Such effects may contribute to lipid and glucose metabolism in peripheral tissues, maintenance of intestinal homeostasis, and modulation of inflammatory status (Niu et al., 2024). The genus Blautia, especially *Blautia wexlerae*, shows a negative correlation with obesity and T2D. Oral administration of *Blautia wexlerae* has been associated with metabolic changes and anti-inflammatory effects in mice, reducing the risk of HFD-induced obesity and diabetes. Its benefits are mediated through unique amino acid metabolic pathways—producing S-adenosylmethionine, acetylcholine, and L-ornithine—and carbohydrate metabolism, which results in the buildup of branched-chain starch and the generation of succinate, lactate, and acetate (Hosomi et al., 2022). Acetatifactor can produce SCFAs, especially acetate and butyrate, which are vital energy sources for intestinal epithelial cells. These metabolites help strengthen the intestinal barrier, reduce inflammation, and influence host metabolism (Pfeiffer et al., 2012; Shin et al., 2024). The abundance of Faecalibaculum is linked to weight loss and improved insulin sensitivity, possibly through the regulation of SCFAs production. Overall, emerging evidence suggests that several commonly used antidiabetic agents are associated with alterations in gut microbiota, multiple hypoglycaemic agents (e.g., metformin, acarbose, and DPP-4is, as mentioned in the introduction) have been linked to improved glycaemic control by modulating these gut microbiota. Our results showed that both dorzagliatin and TTP399 are associated with elevated relative abundance of bacteria such as Akkermansia, Blautia and et al. Previous studies have confirmed these bacteria are closely associated with glucose homeostasis regulation. Based on these observations, we propose that the part hypoglycemic effects of GKAs, similar to metformin, may be linked to direct or indirect interactions with the gut microbiota. There were no systematic studies reported the association between GKAs currently in clinical research phases (including dorzagliatin and TTP399) and gut microbiota. Our research fills this gap.

In addition, our results showed these two GKAs cause different changes in bacterial genera. As a dual hepatic/pancreatic GKA, dorzagliatin promotes the secretion of the GLP-1 (Chen et al., 2023). Previous studies have confirmed that GLP-1 RAs can regulate gut microbiota composition, a systematic review encompassing 38 studies summarized how the GLP-1 RAs improve metabolic homeostasis through gut microbiota regulation (Gofron et al., 2025). A study has indicated that GLP-1 can suppress T cell-mediated inflammatory responses by activating GLP-1 receptors on intestinal intraepithelial lymphocytes (IELs), thereby improving the intestinal immune microenvironment and regulating gut microbiota composition (Wong et al., 2022). Moreover, GLP-1 modulates gastrointestinal motility and secretion (Wettergren et al., 1993; Nauck et al., 1997), which alters the intestinal microenvironment and indirectly modulates gut microbiota composition. In summary, dorzagliatin may alter gut microbiota composition directly or indirectly by promoting GLP-1 secretion. In contrast, TTP399 is a liver-selective GK activator with no significant effect on GLP-1 secretion. This differential regulation of GLP-1 secretion may cause the distinct alterations in bacterial genera induced by these two GKAs. Furthermore, the vehicle1 contains 40% polyethylene glycol (PEG) 300, previous study has demonstrated that PEG 400 can regulate gut microbiota composition in HFD mice (Ishibashi et al., 2023), we hypothesise that PEG 300 may exert similar gut microbiota-regulating effects. The vehicle2 contains 15% Cremophor EL, to date, no studies have investigated the direct effect of Cremophor EL on the gut microbiota. However, similar emulsifiers (e.g., polysorbate 80 (P80) and carboxymethylcellulose (CMC)) have been demonstrate to alter gut microbiota composition (Chassaing et al., 2017). As a similar surfactant, we hypothesise that Cremophor EL has a similar modulatory effect on the gut microbiota. Therefore, the different vehicles of these two GKAs may affect the gut microbiota in bacterial genera.

The gut microbiota plays a role in maintaining intestinal barrier homeostasis and regulating inflammatory responses (Tilg and Moschen, 2014; Han and Lin, 2014; Udayappan et al., 2014; Bäckhed et al., 2004; Wang et al., 2020; Wu et al., 2020). Dysbiosis of the gut microbiota can impair intestinal barrier function, promote systemic inflammation and insulin resistance, and thus worsen metabolic disturbances and the progression of T2D. Such dysbiosis may also weaken the intestinal barrier and decrease the expression of tight junction proteins. Studies have shown that feeding mice a HFD leads to a significant reduction in the levels of tight junction proteins like claudins, occludin, and ZO-1 (Genser et al., 2018; Oliveira et al., 2019). Our results showed that the levels of Tjp1, occludin, and claudin-1 in the HFD groups were not significantly different from those in the CD groups, and we evaluated intestinal inflammatory markers, the results showed that dorzagliatin and TTP399 did not significantly influence intestinal inflammation in mice. In this study, both HFD intervention and drug treatment are short, which may not have been sufficient to induce marked intestinal barrier impairment or to reveal evident improvement effects. So we hypothesize that this may be attributed to the short duration of HFD feeding and GKAs’ treatment, as intestinal barrier function in early-stage HFD-fed mice was still intact, leading to no major changes in tight junction gene expression. Future studies will use a long-term HFD model and prolonged GKAs intervention, to more comprehensively evaluate the regulatory effects of GKAs on intestinal barrier function and local inflammatory markers.

The current study has several limitations. First, this study did not examine changes in gut microbiota metabolites in HFD-fed mice after treatment with dorzagliatin or TTP399. Fecal metabolomics analysis constitutes a crucial research methodology for investigating the functional role of the gut microbiota and the characteristics of its metabolites. This experiment can accurately analyze alterations in the gut microbiota metabolic profile following GKAs intervention, further exploring the potential association between microbial changes and improved glucose metabolism in HFD mice. Moreover, it is critical for elucidating the mechanism by which GKAs ameliorate metabolic disorders through the regulation of the gut microbiota. Second, 4 weeks of drug treatment only reflects the short-term regulatory effects of GKAs on the gut microbiota, cannot evaluate the long-term adaptability of gut microbiota. Third, the intestinal barrier integrity was only evaluated based on histological observations and mRNA expression levels of tight junction–related genes, we did not directly measure intestinal permeability (such as FITC-dextran permeability measurements) and did not validate at the protein level. Based on the current data from this study, we cannot definitively distinguish whether the observed gut microbiota alterations represent direct pharmacological effects of dorzagliatin and TTP399 on the gut microbiota or indirect secondary consequence of improved glycemic control. On one hand, GKAs have hypoglycemic effects, improved glycemic control may alter intestinal nutrient supply, energy status, and intestinal barrier function, thereby indirectly influencing microbial composition. On the other hand, we cannot exclude the possibility that GKAs may directly influence gut microbial growth independently of their hypoglycemic effects. The observed microbiota alterations in this study may result from both direct and indirect effects, and these hypotheses require further investigation. To address these limitations, future studies will integrate long-term GKAs intervention with dynamic monitoring of gut microbiota succession patterns. This will be supplemented by fecal microbiota transplantation and fecal metabolomics (including targeted quantitative analysis of key metabolites in feces, such as SCFA and BA), FITC-dextran permeability measurements and systemic inflammatory markers testing, and we also plan to conduct Western blot or immunohistochemical analyses in future long-term experiments to comprehensively elucidate changes in intestinal barrier function and Inflammation. In subsequent long-term, in-depth research, we plan to refine these experimental components to address the limitations of this study, enhancing completeness and rigor of the study.

In conclusion, this study evaluated the effects of two GKAs, dorzagliatin and TTP399, on the gut microbiota of HFD-fed mice. Our results showed that dorzagliatin raised the relative abundance of several genera, including Akkermansia, Bacteroides, Rikenella, Romboutsia, and Alistipes, while TTP399 increased the relative abundance of Blautia, Acetatifactor, Faecalibaculum, and Bacteroides. These genera have been linked to metabolic homeostasis in previous studies. The hypoglycemic effects of both GKAs may be associated with alterations in the gut microbiota, in the absence of significant changes in intestinal barrier integrity or intestinal inflammation. These findings improve our understanding of the relationship between GKAs, host physiology, and gut microbiota composition, and provide a basis for further mechanistic studies of GKAs.

## 5 Conclusion

In summary, our study further confirms the hypoglycemic effects of dorzagliatin and TTP399 as novel antidiabetic agents, with dorzagliatin also demonstrating effectiveness in improving HFD-induced insulin resistance. Our findings indicate that dorzagliatin and TTP399 are associated with alterations in gut microbiota composition at the genus level in HFD-fed mice, accompanied by increased abundance of some generas that have been linked to metabolic homeostasis in previous studies, with no significant changes observed in intestinal barrier integrity or inflammation. These results highlight a potential association between alterations in gut microbiota and the hypoglycemic effects of dorzagliatin and TTP399, providing novel insights into their mechanisms of action.

## Data Availability

The datasets presented in this study can be found in online repositories. The names of the repository/repositories and accession number(s) can be found at: http://www.ncbi.nlm.nih.gov/bioproject/1431195, PRJNA1431195.

## Glossary

GKAs: Glucokinase activators
GK: Glucokinase
HFD: High-fat diet
T2D: Type 2 diabetes
DPP-4is: Dipeptidyl peptidase-4 inhibitors
GLP-1RAs: Glucagon-like peptide-1 receptor agonists
GIP/GLP-1RAs: Gastric inhibitory polypeptide/glucagon-like peptide-1 receptor agonists
GPCRs: G protein-coupled receptor agonists
LPS: Lipopolysaccharide
AMPK: AMP-activated protein kinase
Tjp1: Tight junction protein 1
SCFA: Short-chain fatty acid
PPAR-*γ*: Peroxisome proliferator-activated receptor-γ
SPF: Specific pathogen-free
FBG: Fasting blood glucose
OGTT: Oral glucose tolerance test
ITT: Insulin tolerance test
AUC: Area under the curve
HOMA-IR: Homeostasis model assessment of insulin resistance
qPCR: Quantitative polymerase chain reaction
PFA: Paraformaldehyde
H&E: Haematoxylin and eosin
SE: Standard error
ANOVA: Analysis of variance
FINS: Fasting insulin
NMDS: Non-metric multidimensional scaling
PCoA: Principal coordinates analysis
LEfSe: Linear discriminant analysis effect size
TG: Triglyceride
TC: Total cholesterol
T1D: Type 1 diabetes
BA: Bile acids
ER: Endoplasmic reticulum
IELs: Intraepithelial lymphocytes
PEG: Polyethylene glycol
P80: P80
CMC: Carboxymethylcellulose
GKRP: GK regulatory protein
GSIS: Glucose-stimulated insulin secretion
