# Supplementary material for: Impact of glucokinase activators on the gut microbiota of high-fat diet-induced obese and type 2 diabetic mice
Source: Front Microbiol. 2026 Mar 19;17:1783385. doi: 10.3389/fmicb.2026.1783385 (PMC13044102; doi:10.3389/fmicb.2026.1783385)
Supplement: Supplementary file 1 [file Table_1.docx]

Supplementary Material

**Supplementary Table 1.** Primer sequence.

| Gene name | Primer sequence |
| --- | --- |
| Tjp1 | F: GCTTTAGCGAACAGAAGGAGC  R: TTCATTTTTCCGAGACTTCACCA |
| Claudin-1 | F: GGGGACAACATCGTGACCG  R: AGGAGTCGAAGACTTTGCACT |
| Occludin | F: TTGAAAGTCCACCTCCTTACAGA  R: CCGGATAAAAAGAGTACGCTGG |
| TNF-α | F: CCA ACG GCA TGG ATC TCA AAGACA  R: AGA TAGCAAATC GGC TGACGG TGT |
| IL-1β | F: TACCTGTGTCTTTCCCGTGG  R: TTGTTCATCTCGGAGCCTGT |
| IL-6 | F: GAGGATACCACTCCCAACAGACC  R: AAGTGCATCATCGTTGTTCATACA |

**
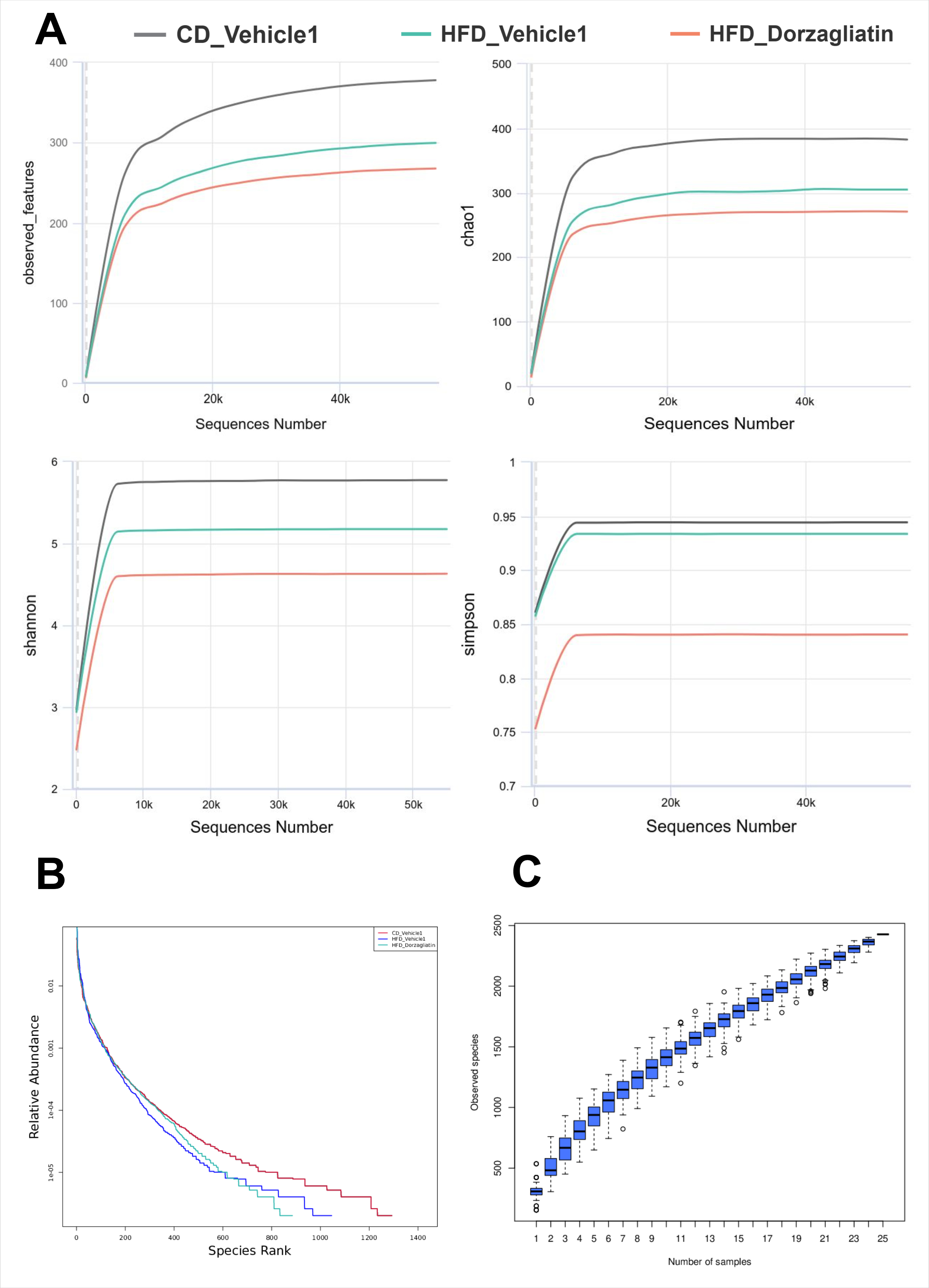
**

**Supplementary Figure 1.** The rarefaction curves (A), species abundance curves (B), and box plots (C). CD_Vehicle1 group: n=7, HFD_Vehicle1 group: n=9, HFD_Dorzagliatin group: n=9.


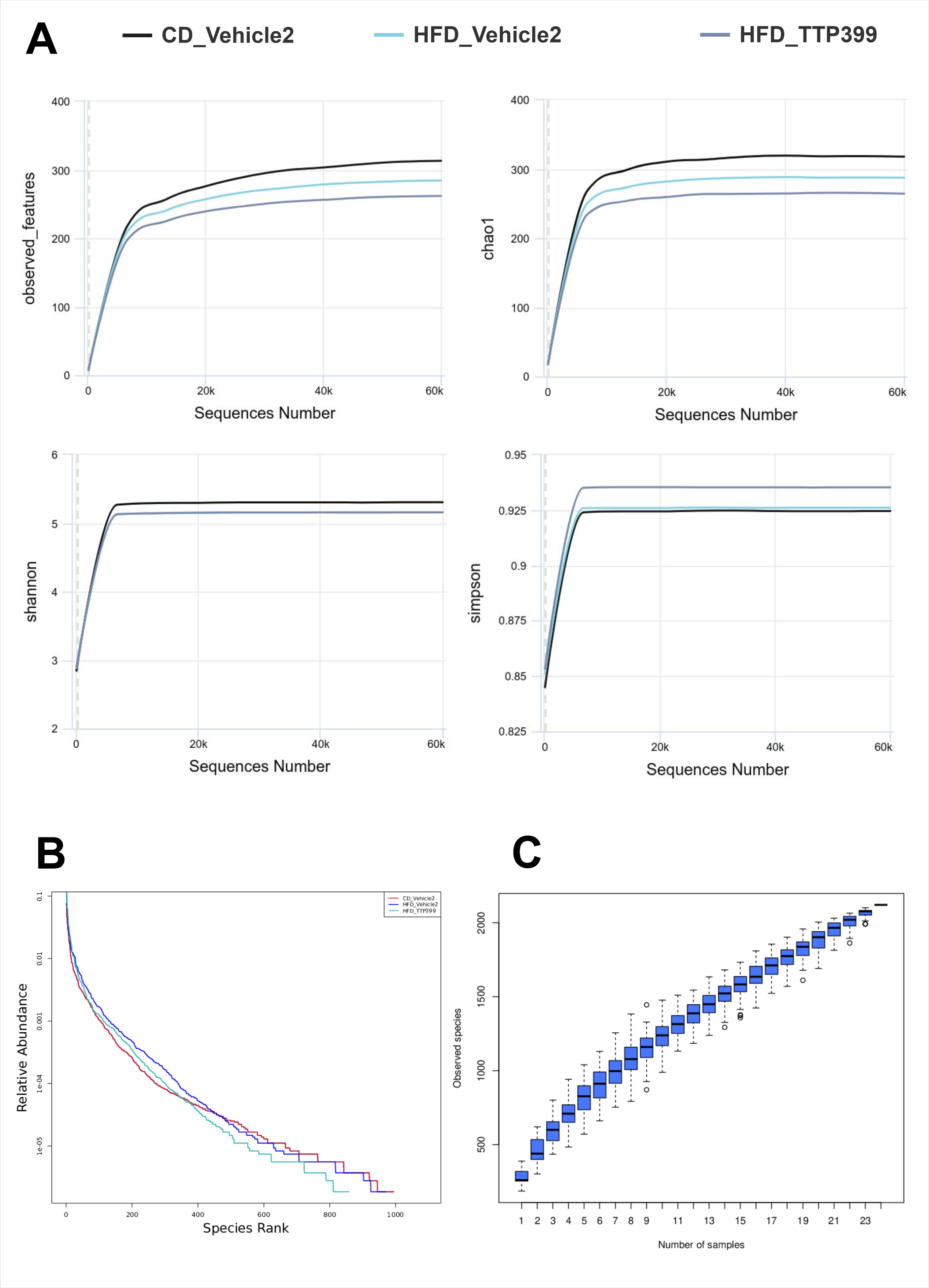


**Supplementary Figure 2.** The rarefaction curves (A), species abundance curves (B), and box plots (C). CD_Vehicle2 group: n=6, HFD_Vehicle2 group: n=9, HFD_TTP399 group: n=9.


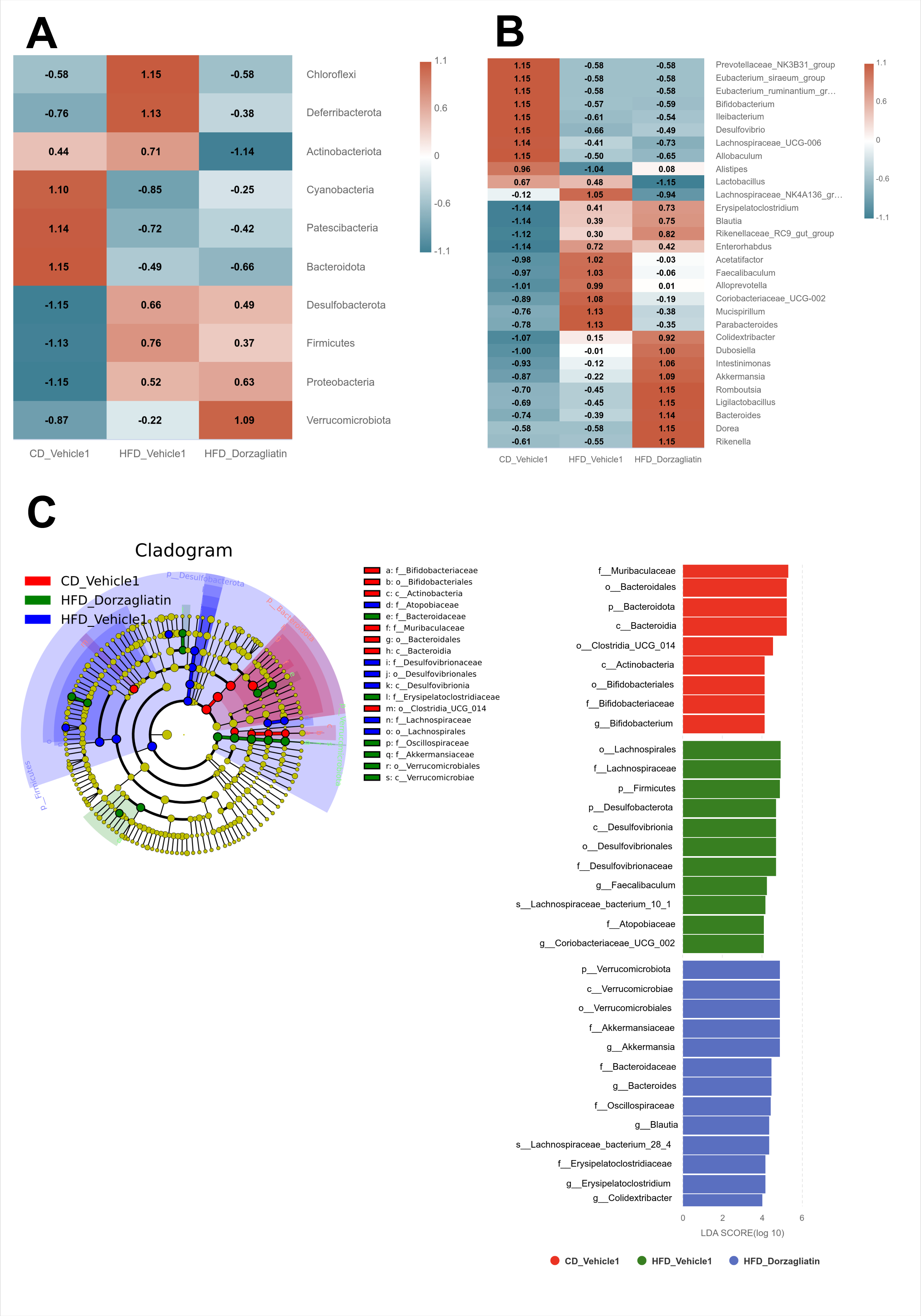


**Supplementary Figure 3.** Effects of dorzagliatin on the composition of the gut microbiota in HFD-fed mice. (A) Relative abundance of the gut microbiota at the phylum levels. (B) Relative abundance of the gut microbiota at the genus levels. (C) Linear discriminant analysis effect size (LEfSe). All data are presented as mean ± SE (CD_Vehicle1 group: n=7, HFD_Vehicle1 group: n=9, HFD_Dorzagliatin group: n=9).


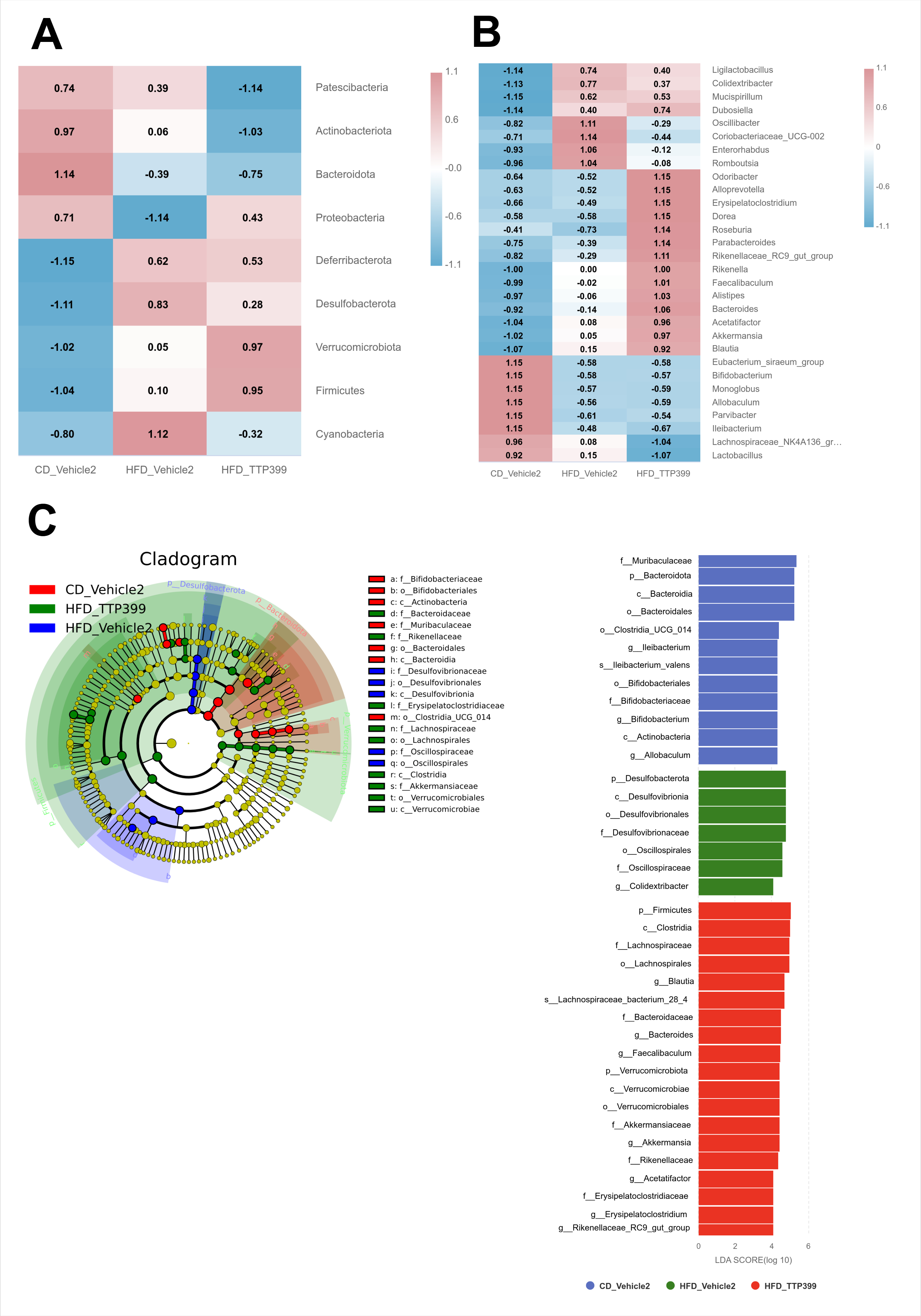


**Supplementary Figure 4.** Effects of TTP399 on the composition of the gut microbiota in HFD-fed mice. (A) Relative abundance of the gut microbiota at the phylum levels. (B) Relative abundance of the gut microbiota at the genus levels. (C) Linear discriminant analysis effect size (LEfSe). All data are presented as mean ± SE (CD_Vehicle2 group: n=6, HFD_Vehicle2 group: n=9, HFD_TTP399 group: n=9).


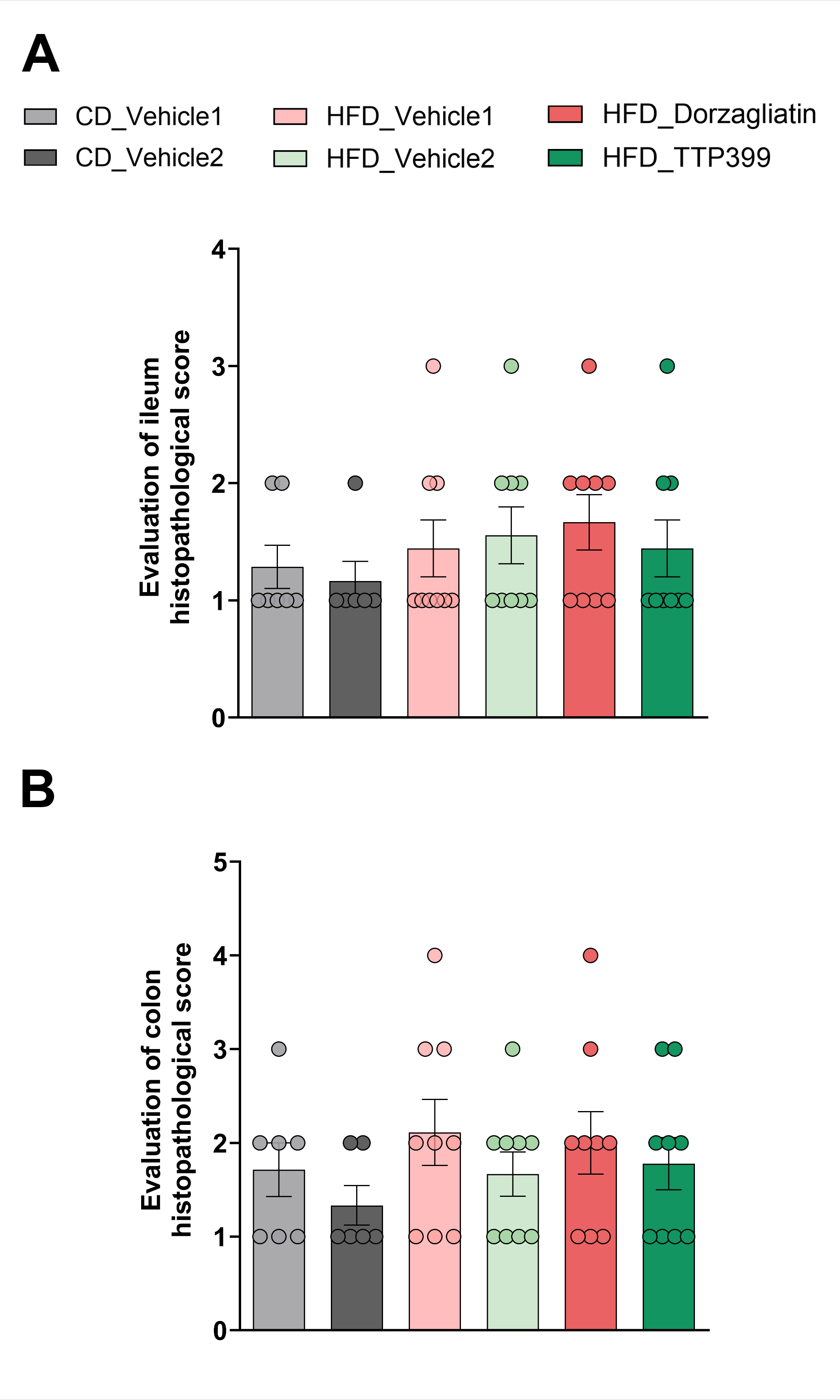


**Supplementary Figure 5.** Dorzagliatin and TTP399 have no significant effect on intestinal barrier integrity or inflammation in HFD-fed mice. (A) Histopathological scores were determined by histopathology Scoring criteria of ileum. (B) Histopathological scores were determined by histopathology Scoring criteria of colon. All data are presented as mean ± SE (CD_Vehicle1 group: n=7, CD_Vehicle2 group: n=6, HFD_Vehicle1 group: n=9, HFD_Vehicle2 group: n=9, HFD_Dorzagliatin group: n=9, HFD_TTP399 group: n=9)
